# MEVDT: Multi-modal event-based vehicle detection and tracking dataset

**DOI:** 10.1016/j.dib.2024.111205

**Published:** 2024-12-09

**Authors:** Zaid A. El Shair, Samir A. Rawashdeh

**Affiliations:** Department of Electrical and Computer Engineering, University of Michigan-Dearborn, 4901 Evergreen Rd, Dearborn, 48128 MI, USA

**Keywords:** Event-based vision, Object detection, Object tracking, Multimodal, Computer vision

## Abstract

In this data article, we introduce the Multi-Modal Event-based Vehicle Detection and Tracking (MEVDT) dataset. This dataset provides a synchronized stream of event data and grayscale images of traffic scenes, captured using the Dynamic and Active-Pixel Vision Sensor (DAVIS) 240c hybrid event-based camera. MEVDT comprises 63 multi-modal sequences with approximately 13k images, 5M events, 10k object labels, and 85 unique object tracking trajectories. Additionally, MEVDT includes manually annotated ground truth labels — consisting of object classifications, pixel-precise bounding boxes, and unique object IDs — which are provided at a labeling frequency of 24 Hz. Designed to advance the research in the domain of event-based vision, MEVDT aims to address the critical need for high-quality, real-world annotated datasets that enable the development and evaluation of object detection and tracking algorithms in automotive environments.

Specifications Table

**Table:** 

Subject	Computer Vision and Pattern Recognition, Computer Science Applications, Signal Processing, Artificial Intelligence
Specific subject area	Event-Based and Multi-Modal Object Detection and Tracking.
Type of data	2D-Grayscale Images (.png), Event Streams (.csv), Fixed-duration Event Files (.aedat), Sequence Annotations (.txt), Sample Annotations (.txt), Train-Test Split Files (.csv)
Data collection	Data was collected using the hybrid sensor DAVIS 240c, which combines an Active Pixel Sensor (APS) and a Dynamic Vision Sensor (DVS) within the same pixel array. The APS captures grayscale images at 24 FPS, while the DVS records pixel brightness changes (*i.e.*, events) at microsecond resolution. The collection process took place at the University of Michigan-Dearborn campus, in two scenes under clear daylight conditions. Data recording was managed using the Robot Operating System (ROS) DVS package running on a laptop. The camera was fixed capturing moving vehicles which were manually labeled with 2D bounding boxes and unique IDs.
Data source location	Institution: University of Michigan-Dearborn City: Dearborn State: Michigan Country: United States of America
Data accessibility	Repository name: Deep Blue Data Data identification number: 10.7302/d5k3-9150 Direct URL to data: 10.7302/d5k3-9150
Related research article	Z. El Shair, S. A. Rawashdeh, High-Temporal-Resolution Object Detection and Tracking Using Images and Events, Journal of Imaging 8 (8) (2022): 210. 10.3390/jimaging8080210 [1]

## 1 Value of the Data

• The MEVDT dataset facilitates the development of models specifically tailored for event-based vision, a cutting-edge imaging technology inspired by the human retina [2]. This dataset provides high temporal resolution and asynchronous event data, essential for capturing dynamic changes in a scene, thereby advancing research in event-based computer vision (CV) tasks such as object detection and tracking.
• Event-based vision is a novel visual sensing modality that offers distinct advantages over conventional frame-based modality, including high dynamic range and robustness to motion blur [2]. MEVDT is one of the few event-based datasets for object detection [[3], [4], [5]] and one of the very few multi-object tracking datasets publicly available [6,7].
• MEVDT provides comprehensive annotations for object tracking. Unlike many existing datasets [6,8,9], MEVDT includes detailed annotations with 2D bounding boxes and unique object IDs. These comprehensive labels are crucial for developing and evaluating object-tracking algorithms, making this dataset a valuable resource for researchers working on high-speed perception and tracking applications.
• By combining asynchronous events from a DVS with synchronous grayscale frames from an APS, the MEVDT dataset supports research into multi-modal data fusion. This capability is important for enhancing the accuracy and robustness of computer vision systems, particularly in challenging conditions where traditional vision systems may struggle.
• Researchers and practitioners in the field of computer vision, specifically within the domain of event-based vision, can utilize this dataset to develop event-based and multi-modal solutions for object detection and tracking, particularly for vehicle-type objects in traffic surveillance scenarios. The high-temporal-resolution and asynchronous event data make this dataset especially suited for applications requiring rapid detection and tracking of moving vehicles, such as real-time traffic monitoring and vehicle flow analysis. Additionally, the provided test set can be used for evaluating the performance and robustness of models designed for high-speed perception in dynamic environments.

## 2 Background

Event-based vision represents a paradigm shift in visual sensing technology, where sensors inspired by the human retina capture dynamic changes in a scene at high temporal resolutions [2,10]. Unlike traditional frame-based cameras, event-based sensors detect per-pixel brightness changes, offering advantages in dynamic range and temporal resolution [2]. This emerging field necessitates specialized datasets to promote research and development, particularly in the CV tasks of object detection and tracking.

Object detection and tracking are critical in various applications like AD and traffic monitoring [1,11]. Event cameras, with their low latency and high-temporal-resolution output, offer promising prospects in these areas. However, the development of relevant methodologies has been impeded by a lack of labeled event-based datasets tailored to these applications. Existing datasets often lack the necessary annotations, such as object IDs, essential for tracking applications [[3], [4], [5],^8^,^9^,^12^].

To address this limitation, we have created a dataset specifically tailored for event-based and multi-modal object detection and tracking. MEVDT includes sequences with multiple vehicles moving at various speeds, featuring manually labeled bounding boxes and object IDs, which are vital for enabling object tracking evaluation.

In contrast to our prior publications [1,13], which presented methods that utilize this dataset, this article provides a comprehensive overview and in-depth analysis of the MEVDT dataset itself. This article offers a detailed breakdown of the dataset's statistics and introduces a sequence-based training/testing split, facilitating its use in model development and evaluation.

## 3 Data Description

This section outlines the MEVDT datasetʼs structure, offering detailed insights into its organization and content to support various CV tasks. First, we describe the main statistics of the dataset and detail each sequenceʼs characteristics (Section 3.1). Then, we present the datasetʼs directory structure by outlining each subdirectoryʼs purpose and contents (Section 3.2). Finally, we detail the different data sample formats (Section 3.3) and label formats (Section 3.4) available in MEVDT.

### 3.1 Dataset statistics

The MEVDT dataset contains multiple recordings of different vehicles moving at varying speeds captured at two different scenes (Scene A and Scene B). These recordings are segmented into shorter sequences for a more focused analysis and usage. Accordingly, Scene A includes 32 sequences, comprising 9274 images and 6828 annotations, while Scene B contains 31 sequences with 3485 images and 3063 annotations. Additionally, each sequence includes a continuous stream of asynchronous events captured by the event camera. Overall, our dataset provides a total of 9891 vehicle annotations. The discrepancy between the number of images and annotations arises from frames without detectable objects.

The sequence statistics for each scene, including sequence durations, number of images, events, and objects, are summarized in Table 1. On average, each generated sequence is approximately 9 s in length with around 200 images and 87,000 events. This translates to an average event rate of 10,000 events per second, underscoring the high temporal resolution characteristic of event-based sensors. As a result of our labeling, the dataset provides 85 different continuous object trajectories in total, each represented by a unique object ID.

**Table 1:** Sequence statistics for Scenes A and B in the MEVDT dataset. This table details the total number of sequences for each scene and provides the total and average (with standard deviations) sequence duration, number of images, events, objects, object IDs, and average bounding box area.

Subset	# of Seqs.	Seq. Duration (*s*)		# of Images		# of Events		# of Objects		# of Object IDs		Average BB Area (*pixel*^2^)
		Total	Average ± SD	Total	Average ± SD	Total	Average ± SD	Total	Average ± SD	Total	Average ± SD	
Scene A	32	397.3	12.42 ± 9.94	9274	289.81 ± 230.8	2,269,913	70,935 ± 59,337	6828	213 ± 147	54	1.7 ± 1.1	1960.5
Scene B	31	147.7	4.76 ± 3.55	3485	112.42 ± 82.4	3,195,652	103,086 ± 31,950	3063	99 ± 84	31	1 ± 0	4093.2
**Total**	63	545.0	8.65 ± 8.39	12,759	202.52 ± 194.7	5,465,565	86,755 ± 50,169	9891	157 ± 132	85	1.3 ± 0.8	3010.0

Additionally, we provide sequence-based training and test splits. These splits are critical for methodical model development and evaluation, ensuring reproducibility and consistency in experimental setups. We allocate approximately 80 % of the sequences for training and 20 % for testing in both Scene A and Scene B, ensuring a well-balanced distribution of images, events, and objects. This balance is achieved by establishing an appropriate 80–20 % (4:1) distribution across all of the datasetʼs parameters. Details of this distribution are demonstrated in Table 2, Table 3 for Scene A and Scene B, respectively. The 80–20 split, a standard heuristic in machine learning, strikes a balance between the need for ample training data and representative testing data, promoting robust model training, preventing overfitting, and enabling reliable model validation and generalization to unseen data.

**Table 2:** Training and testing split statistics for Scene A. This table provides a comprehensive breakdown of the sequences in this location, including their durations, number of images, events, objects, object IDs, and average bounding box areas for both training and testing subsets.

	# of Seqs.	Seq. Duration (*s*)	# of Images	# of Events	# of Objects	# of Object IDs	Average BB Area (*pixel*^2^)
**Training**							
Average	*—*	12.2	284.0	72,512.3	210.8	1.7	1932.7
SD	*—*	9.8	227.4	63,088.0	138.8	1.0	914.8
Total	26	316.4	7385	1,885,319	5481	44	50,249.8
%	81 %	80 %	80 %	83 %	80 %	81 %	80 %
**Testing**							
Average	*—*	13.5	314.8	64,099.0	224.5	1.7	2081.1
SD	*—*	9.6	224.1	29,780.6	166.1	1.1	1109.4
Total	6	80.9	1889	384,594	1347	10	12,486.5
%	19 %	20 %	20 %	17 %	20 %	19 %	20 %

**Table 3:** Training and testing split statistics for Scene B. This table provides a comprehensive breakdown of the sequences in this location, including their durations, number of images, events, objects, object IDs, and average bounding box areas for both training and testing subsets.

	# of Seqs.	Seq. Duration (*s*)	# of Images	# of Events	# of Objects	# of Object IDs	Average BB Area (*pixel*^2^)
**Training**							
Average	—	4.7	109.9	103,113.4	96.0	1	4088.3
SD	—	3.3	76.4	32,434.7	77.1	0	1123.9
Total	25	116.3	2747	2,577,836	2400	25	102,208.1
%	81 %	79 %	79 %	81 %	78 %	81 %	81 %
**Testing**							
Average	—	5.2	123.0	102,969.3	110.5	1	4113.5
SD	—	4.2	97.5	26,843.0	99.9	0	1011.2
Total	6	31.3	738	617,816	663	6	24,681.1
%	19 %	21 %	21 %	19 %	22 %	19 %	19 %

Detailed breakdown of each sequence in Scene A and Scene B are demonstrated in Table 4, Table 5, respectively. These breakdowns provide detailed information on each sequence, including the sequence name (identified by the first data timestamp in nanoseconds), duration, number of images, events, objects, and the average area of bounding boxes, along with their allocation to either training or testing splits. This detailed information aids in understanding the dataset composition and its distribution between training and testing.

### 3.2 Dataset structure

The MEVDT dataset is structured into several primary directories, each tailored to specific data handling needs:

• **sequences/**: Stores sequences of images and corresponding event streams, serving as the primary data source for object-tracking solutions.
• **labels/**: Contains detailed ground truth labels for object detection and object tracking, essential for model training and evaluation.
• **event_samples/**: Features fixed-duration event-data samples extracted at different batch-sampling durations, providing varied temporal resolutions for advanced event-based methodologies.
• **data_splits/**: Includes Comma-Separated Values (CSV) files that index the path of each sample, including the extracted event sample file (provided in event_samples/), corresponding image (available in sequences/), and detection label file (within labels/detection_labels/) facilitating straightforward data loading and partitioning.

**Fig. 1:**
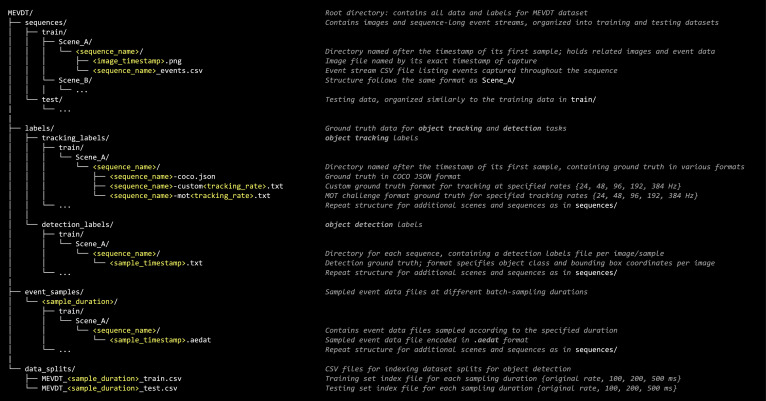
MEVDT dataset directory structure and organization.

Generally, the dataset is split into training and testing splits. Within each split, a list of sequences per the locations Scene_A and Scene_B is provided. The full dataset structure and directory breakdown are detailed in Fig. 1.

### 3.3 Sample formats

The dataset samples are provided in various formats. As shown in Fig. 1, the grayscale images are stored in a standard Portable Network Graphics (PNG) format. Meanwhile, the sequence-long event streams are provided in a CSV file where each line corresponds to a single event in a comma-separated format as follows:

(1) $$\begin{array}{c} \mathrm{ts},\;x,\;y,\;p\;, \end{array}$$

where ts denotes the timestamp of the event in nanoseconds, while x and y correspond to the two-dimensional (2D) pixel coordinate at which the event has occurred. Finally, p denotes the polarity of the event as either positive (p=1) or negative (p=0).

In contrast, the extracted event samples, provided in the event_samples/ directory, are stored in a different format. These samples, generated at various fixed sampling durations, are encoded in *AEDAT* 3.1 file format following the *DVS-Gesture* dataset's implementation [14].

### 3.4 Label formats

The MEVDT dataset includes separate ground truth label files for both object detection and object tracking. Each label format is provided in their respective directories within labels/.

#### 3.4.1 Object tracking

As demonstrated in Fig. 1, the object tracking label files are available in three formats:

• COCO annotations in JSON file format [15].
• MOT Challenge format [16].
• Custom format.

The COCO format is well-defined in [15]. The MOT Challenge format, detailed in [16], is necessary for using *TrackEval* [17], which facilitates the generation of multi-object tracking (MOT) results as demonstrated in [1,13]. Our custom format, which utilizes a single line per sample, is defined as follows:

(2) $$\begin{array}{c} \mathrm{frame}\_\mathrm{id},\;\mathrm{frame}\_\mathrm{ts},{\left\{\mathrm{obj}\_id_{i},\;x_{i},\;y_{i},\;w_{i},\;h_{i}\right\}}_{i=1}^{n}\;, \end{array}$$

where frame_id is the frame's unique identifier within the sequence and frame_ts is the frameʼs timestamp in nanoseconds. Each set, denoted by $\{\mathrm{obj}\_id_{i}$, $x_{i}$, $y_{i}$, $w_{i}$, $h_{i}\}$, specifies the bounding box for the ith object, detailing its identifier $\mathrm{obj}\_id_{i}$, top-left corner coordinates $(x_{i}$, $y_{i})$, width $w_{i}$, and height $h_{i}$ when present in the given frame. This format supports an arbitrary number of objects per frame and uses nanosecond timestamps to ensure synchronization with high-temporal-resolution event data. We also provide ground truth for multiple tracking rates (24, 48, 96, 192, and 384 Hz) in both our custom and MOT Challenge [16] formats.

#### 3.4.2 Object detection

Object detection labels, as illustrated in Fig. 1, consist of a label file per sample. The label format for each object detected in a sample is represented as follows:

(3) $$\begin{array}{c} \mathrm{class}\_\mathrm{idx}\;x\_\min\;y\_\min\;x\_\max\;y\_\max\;, \end{array}$$

where class_idx denotes the object's category (set as 1 for the vehicle class). The coordinates (x_min,y_min) and (x_max,y_max) define the top-left and bottom-right corners of the object's bounding box, respectively. Each line within a file represents a separate object; therefore, the label files may contain multiple lines — or none — each corresponding to a single object instance. For example, if a vehicle is labeled within an image with its bounding box starting at (50,50) and extending to (200,150), then the label file would contain the following:

(4) $$\begin{array}{c} 1\;50\;50\;200\;150\;. \end{array}$$

This line indicates a single vehicle, categorized under index 1, occupying the specified pixel range. If multiple vehicles are detected, each will be represented by a similar line within the same file detailing its classification and location. Note that class_idx skips the index 0 as it typically represents the `background` class in most object detectors.

## 4 Experimental Design, Materials and Methods

In this section, we detail the data collection and labeling process used to create the MEVDT dataset, which focuses on event-based vision for vehicle detection and tracking. We begin with an overview of the hardware and sensor specifications (Section 4.1), followed by the data collection setup, including the locations and conditions used (Section 4.2). Finally, we outline the labeling process employed in this work (Section 4.3).

### 4.1 Hardware and sensor specifications

We utilized the hybrid sensor DAVIS 240c,^1^ which combines an APS and a DVS within the same pixel array at a resolution of 240 × 180. This sensor captures both synchronous intensity frames and asynchronous events, providing a comprehensive visual dataset crucial for developing event-based and multi-modal solutions. The APS captures intensity (*i.e.*, grayscale) frames at approximately 24 frames per second (FPS), while the DVS records changes in pixel intensity — known as events — at a microsecond resolution, essential for capturing changes in the scene at very high speeds. The camera does not feature auto-exposure; hence, we manually adjusted the camera lens to ensure proper exposure before initiating the data collection at each location.

Additionally, during a subset of the data collection process, we employed a high-speed LiDAR, Benewake TF03-100, to provide high-temporal-resolution ground truth positional measurements. This LiDAR, capable of delivering measurements at rates up to 1000 Hz, was placed 30 to 60 m from the vehicles and used to precisely estimate distances to the tracked vehicles at high tracking rates. Although these positional measurements offer valuable insights for validating object tracking performance, they are not included in the MEVDT dataset as it is aimed primarily at CV tasks of object detection and tracking. The primary reason for their omission is to maintain the datasetʼs focus on visual data processing challenges, as the LiDAR data falls outside the typical usage scenarios intended for users of this dataset. Detailed insights and applications of this LiDAR data in evaluating tracking accuracy are further explored in our prior work [13].

### 4.2 Data collection setup

**Fig. 2:**
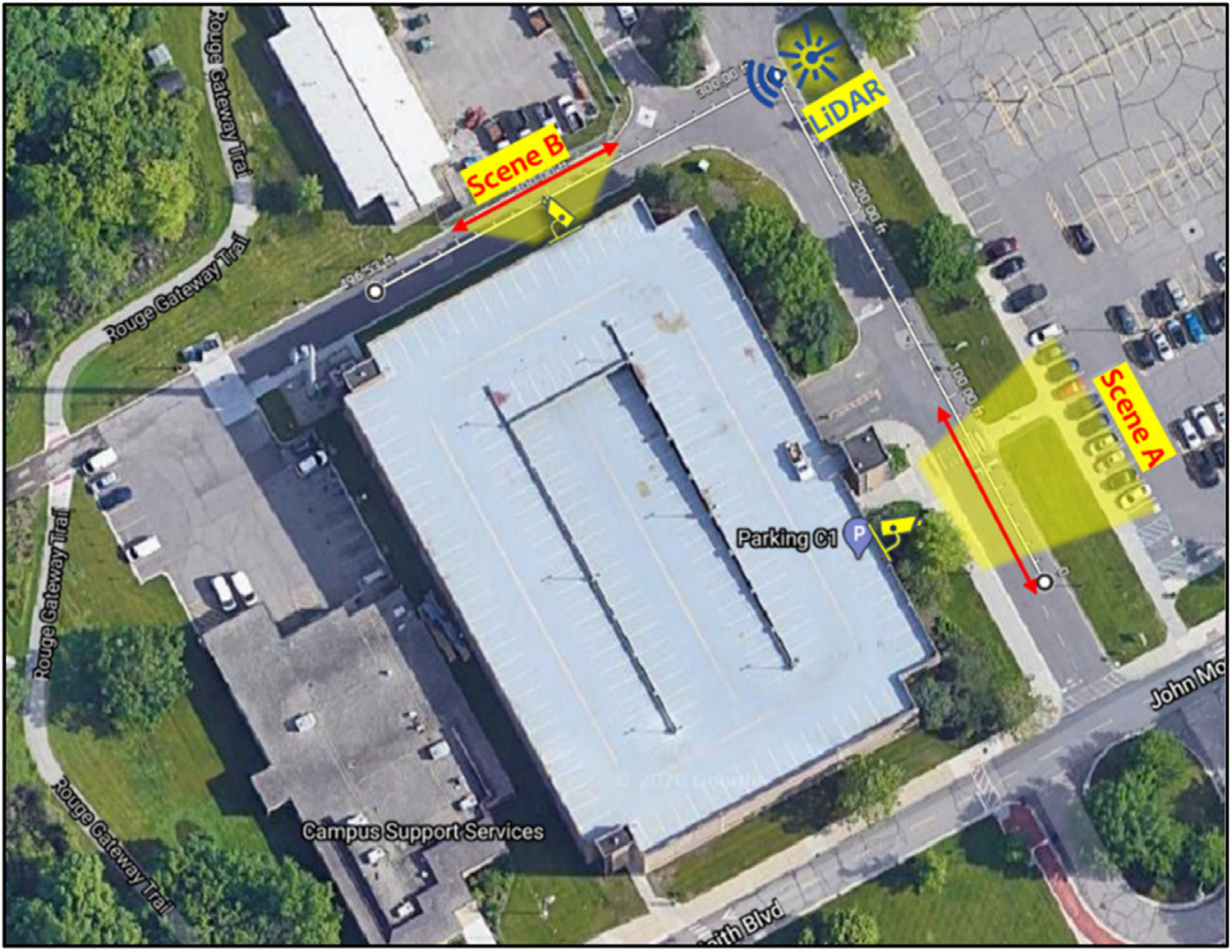
Satellite view of a subsection of the University of Michigan-Dearborn campus highlighting Scene A and Scene B, where data was collected, along with the position of the LiDAR sensor.

**Fig. 3:**
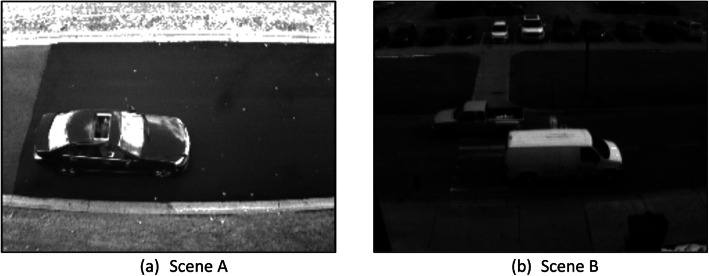
Sample image outputs from the dataset demonstrating the two distinct scenes, including (a) Scene A and (b) Scene B, showcasing the camera's perspective and field of view for each location within the University of Michigan-Dearborn's campus.

Using DAVIS 240c and the ROS DVS package developed by Robotics and Perception Group^2^ [18] for data recording, we collected several hours of spatiotemporally synchronized images and events. The data was collected at two distinct locations on the University of Michigan-Dearborn campus, referred to as Scene A and Scene B. These locations were selected for their proximity to traffic and high-viewing perspective, providing optimal views of moving vehicles. A satellite view, depicting the data collection locations, and a sample image from each scene are shown in Fig. 2, Fig. 3, respectively. Data was recorded on separate days at each scene under stable daylight conditions were selected to minimize lighting variation, ensuring consistent data quality.

**Fig. 4:**
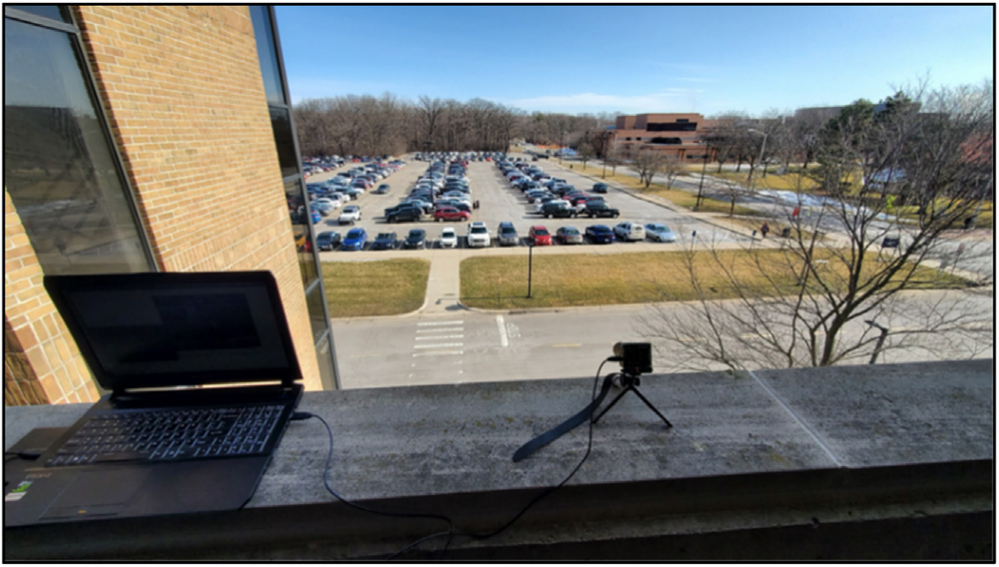
The data collection setup showing the hybrid event camera (DAVIS 240) mounted on a tripod at the edge of a building overlooking the street and part of the parking lot. A laptop adjacent to the camera setup is used for data recording and sensor control.

During the data collection process, the event camera was placed on the edge of the third floor of a parking structure building pointing downward at the street, representing an infrastructure-based traffic surveillance perspective, as demonstrated in Fig. 4. The position of the camera and the utilized lens were manually adjusted to create the fields of view shown in Fig. 3. This static setup ensured that recorded events captured only object motion or noise without introducing camera ego-motion.

**Fig. 5:**
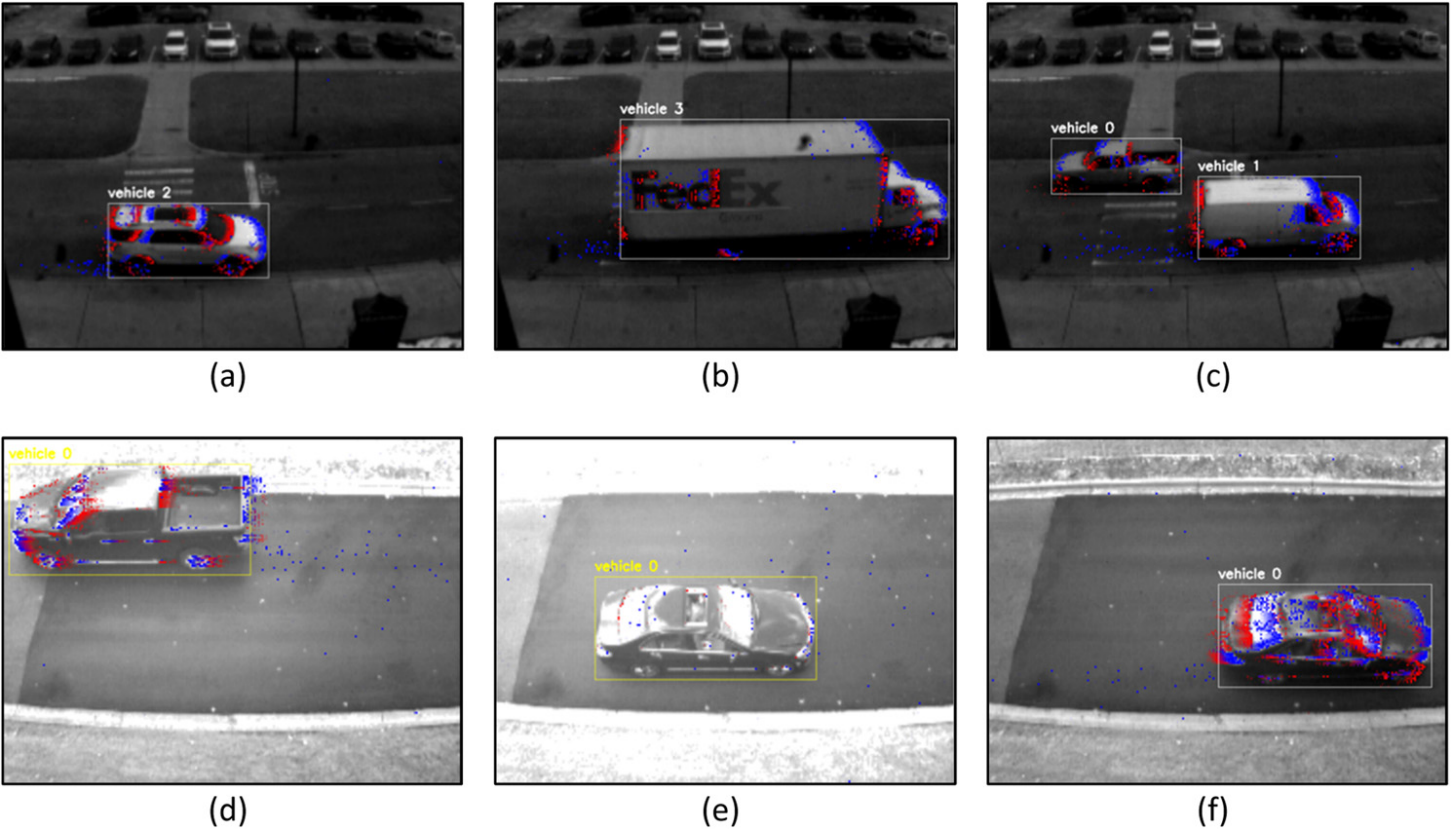
Samples from the dataset showing labeled vehicles. Each image demonstrates the APS intensity frame with superimposed events from the DVS collected in the last ∼43 ms, where blue and red pixels visualize positive and negative events, respectively. The samples include various vehicle types such as (a) SUVs, (b) trucks, (c) vans, and (d) pickup trucks captured in two different scenes (Scene A for the top row and Scene B for the bottom row). The presence of multiple objects and vehicles at different speeds (e–f) illustrates the dataset's utility for object detection and tracking research.

In this dataset, we focused on capturing sequences of moving vehicles of different types (*e.g.*, sedans, trucks), as shown in Fig. 5. While a few sequences in Scene A included pedestrians passing by, these instances were excluded from the annotations process due to their slow movement and distant proximity to the camera, which presented challenges for object detection. This dataset emphasizes vehicle detection and tracking across varying speeds and acceleration rates, with some coming to a full stop, making detection and tracking tasks especially challenging when relying solely on event-based data from the DVS.

### 4.3 Data labeling and processing

Labeling was manually performed for each vehicle detected in the scene using the online *dLabel Annotation Tool.*^3^ This tool was selected for its precision and ease of use in annotating objects for object detection and tracking applications. Further, it supports sub-pixel accuracy in annotations and includes features such as bounding box interpolation across sequences, which is quite useful for annotating unique objects that appear in consecutive images, thereby significantly reducing the effort and time required for manual labeling. Only vehicle-type objects were labeled, with each vehicle instance carefully marked by a 2D bounding box and assigned a unique object ID to ensure tracking continuity using the intensity images captured by the APS. This process resulted in a labeling frequency of ∼24 Hz, matching the framerate of the camera's APS. Annotations were carried out by research team members with relevant experience. Regular quality checks were conducted across sequences to maintain precision. Importantly, no significant errors or issues were encountered during the annotation process.

After the annotation process, the resulting labels were initially saved in the COCO format [15] and subsequently converted to the various formats and temporal resolutions detailed in Section 3.4 using custom scripts developed specifically for this dataset. These scripts utilize the dataʼs microsecond timestamps to interpolate the ground truth labels, thus generating high-temporal-resolution labels that significantly enhance the dataset's utility for high-precision object tracking.

Labels are directly transferable to the event-based modality thanks to the temporal and spatial synchronization between the APS and DVS. The temporal synchronization is enabled by DAVIS's high-resolution clock [19], while the spatial synchronization is facilitated by the shared lens between the camera's APS and DVS. This synchronization ensures that a pixel $\left(x_{i},\;y_{i}\right)$ in one modality precisely corresponds to the same pixel in the other, significantly enhancing the dataset's utility for cross-modal and multi-modal studies by allowing annotations to be used seamlessly across both.

The labeled MOT data provides true 2D bounding boxes for all vehicles in the scene present in any image, along with their corresponding object IDs, essential for proper object tracking evaluation. In contrast, the object detection labels, as detailed in Section 3.4, include the object classification index and 2D bounding box coordinates. Fig. 5 demonstrates several samples from our dataset, showcasing ground truth annotations with objects’ bounding boxes and unique IDs, with the latest ∼43 ms of events superimposed on each grayscale image.

#### 4.3.1 Excluded objects

In the MEVDT dataset, our labeling efforts primarily focused on moving vehicles. Parked vehicles in Scene A, shown in Fig. 3a, were intentionally excluded to maintain the dataset's emphasis on dynamic scenarios. These stationary vehicles, located at the top of the frame, were not labeled due to their static nature and relative size, including any moving vehicles behind them. Users are advised to crop or ignore the upper 15–20 % of the frame in Scene A during training or fine-tuning deep learning-based models to avoid these static vehicles. Alternatively, the detections of the parked vehicles can be disregarded when using off-the-shelf pre-trained object detectors, such as YOLOv3 [20], as implemented in our prior research [1,13].

Additionally, while our labeling efforts concentrated on moving vehicles, some sequences in Scene A do include a few pedestrians. These pedestrians were not labeled due to two reasons. First, the occurrence of pedestrians in the MEVDT dataset is minimal. Only a handful of sequences in Scene A feature pedestrians, making their presence rare and insufficient for substantial analysis. Second, including the few pedestrian annotations would significantly imbalance the dataset, making model training and evaluation less feasible. The focus of MEVDT is on dynamic and prevalent traffic elements, specifically moving vehicles, which are more numerous and varied in the recorded scenes. Future data collection efforts should consider locations with a higher number of active and mobile pedestrians to create a more balanced dataset that includes diverse object categories. This would enable better training and evaluation of models for both vehicles and pedestrians.

## Limitations

Although the MEVDT dataset provides valuable data for event-based vision research, it has certain limitations. The dataset focuses exclusively on vehicles, with no labeled pedestrians, which reduces object variety and may affect the generalizability of models. Due to the proximity of the camera, the object sizes are generally uniform with relatively similar viewing perspectives, though some variations exist between scenes. Additionally, the camera remains fixed throughout the data collection process, resulting in negligible ego motion — an essential aspect for some applications, such as AD. The dataset's scale, though substantial, may be insufficient for training highly complex models or tasks requiring large-scale data. Finally, environmental variations, such as lighting and weather conditions, are limited, potentially impacting the robustness of models in diverse real-world scenarios.

## Ethics Statement

The authors confirm they have read and followed the ethical requirements for publication in Data in Brief and confirm that the current work does not involve human subjects, animal experiments, or any data collected from social media platforms.

## CRediT Author Statement

**Zaid A. El Shair:** Conceptualization, Data curation, Investigation, Methodology, Software, Visualization, Writing; **Samir A. Rawashdeh:** Conceptualization, Methodology, Project administration, Resources, Supervision, Validation.

## Data Availability

Deep Blue DataMEVDT: Multi-Modal Event-Based Vehicle Detection and Tracking Dataset (Original data).

## Appendix

**Table 4:** Detailed sequence statistics for Scene A. This table includes information on each sequence's duration, number of images, events, objects, object IDs, average bounding box area, and allocation to training or testing splits.

Sequence #	Sequence Name	Duration (*s*)	# of Images	# of Events	# of Objects	# of Object IDs	Average BB Area (*pixel*^2^)	Train | Test
1	1,581,956,305,832,790,936	9.5	222	76,924	240	2	1852.4	Train
2	1,581,956,366,514,475,936	21.2	494	48,556	122	1	1562.3	Train
3	1,581,956,422,501,835,936	10.4	243	70,138	241	2	1490.3	Test
4	1,581,956,475,991,297,936	23.3	542	61,664	135	1	1754.2	Test
5	1,581,956,525,690,846,936	17.4	404	79,738	382	2	1476.3	Train
6	1,581,956,568,112,038,936	5.3	124	34,207	117	1	1394.1	Test
7	1,581,956,586,329,463,936	12.1	283	114,163	186	1	2997.0	Train
8	1,581,956,636,804,222,936	4.9	115	29,212	102	1	1522.7	Train
9	1,581,956,672,808,401,936	5.4	127	60,633	118	1	1665.5	Train
10	1,581,957,068,983,574,936	21.0	488	154,064	420	3	3373.5	Train
11	1,581,957,114,204,134,936	7.0	163	34,310	160	1	1609.8	Train
12	1,581,957,156,969,863,936	8.3	195	62,531	192	1	4538.2	Test
13	1,581,957,173,378,467,936	2.5	59	20,295	58	1	1315.7	Train
14	1,581,957,190,648,414,936	45.6	1061	107,768	224	1	1796.1	Train
15	1,581,957,249,133,671,936	6.7	158	51,306	150	1	1730.8	Train
16	1,581,957,506,675,527,936	18.1	421	77,074	502	3	1373.6	Train
17	1,581,957,567,314,145,936	4.1	96	34,093	81	1	1710.7	Train
18	1,581,957,616,841,425,936	10.3	241	41,452	208	2	1386.9	Train
19	1,581,957,903,798,179,936	6.2	145	31,237	130	1	1658.5	Train
20	1,581,957,963,058,646,936	5.2	124	48,102	122	1	2071.8	Train
21	1,581,958,023,266,591,936	4.6	109	20,877	97	1	1476.4	Train
22	1,581,958,094,284,404,936	5.1	119	14,818	113	1	1587.9	Train
23	1,581,958,106,816,959,936	14.9	348	140,138	399	3	1396.0	Train
24	1,581,958,201,263,329,936	25.4	592	91,349	239	3	1737.3	Train
25	1,581,958,201,392,531,936	2.8	65	23,145	64	1	1608.6	Train
26	1,581,958,289,206,551,936	14.9	346	71,577	282	3	1816.2	Train
27	1,581,958,320,817,876,936	5.4	126	76,807	113	1	5773.9	Train
28	1,581,958,380,465,948,936	29.7	694	122,720	578	4	1487.0	Test
29	1,581,958,511,820,908,936	9.7	226	78,420	198	2	1541.7	Train
30	1,581,958,540,632,865,936	3.9	91	33,334	84	1	1822.8	Test
31	1,581,958,551,959,539,936	29.0	676	330,350	605	5	2782.1	Train
32	1,581,958,587,877,583,936	7.6	177	28,911	166	1	1426.3	Train

**Table 5:** Detailed sequence statistics for Scene B. This table includes information on each sequence's duration, number of images, events, objects, object IDs, average bounding box area, and allocation to training or testing splits.

Sequence #	Sequence Name	Duration (*s*)	# of Images	# of Events	# of Objects	# of Object IDs	Average BB Area (*pixel*^2^)	Train | Test
1	1603470885671858364	14.1	329	130,227	321	1	5706.2	Test
2	1603470907722265364	4.9	116	109,775	107	1	4216.0	Train
3	1603470947042618364	8.3	195	106,234	188	1	2468.5	Train
4	1603471304371177364	5.8	137	123,331	90	1	3988.2	Train
5	1603471325344903364	2.1	49	87,050	44	1	3017.5	Train
6	1603471347223041364	2.7	65	106,671	55	1	3995.4	Train
7	1603471362511897364	2.4	58	91,918	47	1	3043.1	Train
8	1603471387318604364	2.5	61	108,368	52	1	3971.9	Train
9	1603471400411033364	2.1	51	64,688	34	1	2924.9	Test
10	1603471419705138364	6.0	142	91,522	127	1	5007.8	Train
11	1603471437405757364	1.7	41	55,782	27	1	3028.5	Train
12	1603471457905745364	6.8	159	92,745	142	1	4610.4	Train
13	1603471475606364364	2.4	56	67,010	40	1	3087.3	Train
14	1603471489904674364	2.9	68	151,037	52	1	4502.9	Train
15	1603471504116850364	6.9	163	94,593	152	1	5330.4	Train
16	1603471523712426364	2.3	56	67,044	45	1	2963.8	Test
17	160347154451388464	17.6	410	104,188	400	1	5260.7	Train
18	1603471574445588364	2.3	56	64,461	43	1	3054.5	Train
19	1603471594816373364	6.8	159	105,311	150	1	5210.7	Train
20	1603471817884627727	2.8	67	83,430	53	1	4643.3	Train
21	1603471844801627727	3.8	91	116,064	76	1	4082.0	Test
22	1603471863492792727	4.1	96	216,927	76	1	7410.9	Train
23	1603471880891941727	3.6	86	103,624	64	1	3000.1	Train
24	1603471908885621727	6.4	151	112,285	142	1	5159.1	Train
25	1603471928136659727	2.5	61	72,175	49	1	3057.4	Train
26	1603471965045249727	6.8	161	119,105	151	1	5128.6	Train
27	1603471985975909727	3.1	73	87,510	47	1	3042.6	Train
28	1603472011687027727	2.7	65	113,585	51	1	3972.8	Test
29	1603472029387646727	2.8	68	74,378	57	1	3059.0	Train
30	1603472052643934727	6.2	146	126,208	136	1	5031.5	Test
31	1603472118493683727	2.0	49	148,406	45	1	3913.5	Train

## Footnotes

1 DAVIS 240c specifications available at https://inivation.com/wp-content/uploads/2019/08/DAVIS240.pdf.

2 Available at https://github.com/uzh-rpg/rpg_dvs_ros.

3 Available at https://dlabel.org/.
